# How to reduce anxiety symptoms through individual sport in youth: A longitudinal study over 8-month observation

**DOI:** 10.1177/20503121241258736

**Published:** 2024-06-17

**Authors:** Lin Wang, Tianle Zhang, Weihao Huang, Leyuan Gou, Ming Zhong, Qiaohan Liu, Yihao Liu

**Affiliations:** 1 Sports Department, Guilin University of Electronic Science and Technology, Guilin, China; 2 Sport and Health Science, Faculty of Life & Environmental, University of Exeter, Exeter, UK; 3 Department of Computer Science, University of Liverpool, Liverpool, UK; 4 School of Psychology, Faculty of Life & Environmental Sciences University of Exeter, Exeter, UK

**Keywords:** Anxiety symptoms, youth, individual sport, longitudinal study, well-being

## Abstract

**Background:**

Anxiety symptoms are widely observed among the youth, and engagement in sports has been demonstrated to mitigate these symptoms. Nonetheless, the effectiveness of specific sports and the potential moderating role of psychological factors, such as self-esteem and self-efficacy, on the influence of sports on anxiety, remains to be elucidated. This study was designed to longitudinally assess the impact of sports participation on anxiety symptoms among young individuals.

**Methods:**

The study encompassed 163 university students, with a male predominance of 81.6%, and explored the influence of sport-related factors (such as mastery of table tennis skills, level and engagement) and psychological aspects (including self-efficacy, self-esteem and resilience) on anxiety symptoms, employing an 8-month longitudinal approach. Physical activity, sedentary and sleep behaviour, along with age, body mass index (BMI), and sex, were accounted for as confounding variables.

**Results:**

The study found that high table tennis performance score was found to buffer the development of anxiety symptoms in students with decreased self-esteem in an exploratory moderation model. Self-esteem and self-efficacy were negatively associated with the development of anxiety symptoms, whereas physical activity factors did not have a direct effect.

**Conclusion:**

This study highlights the potential of table tennis as a form of sport to alleviate anxiety symptoms in university students, particularly among those with decreased self-esteem. Future research should address the study’s limitations and explore the potential moderating effects of other psychological factors. Overall, these findings suggest a potential new approach to treating anxiety symptoms among university students.

## Highlight of this study

Participation in sports has been shown to alleviate symptoms of anxiety in young people. Furthermore, activities like table tennis can have beneficial effects on mental health and well-being. However, there is yet to be evidence demonstrating how individual sports, such as table tennis, contribute to the long-term reduction of anxiety symptoms.

Our research suggests that individual sports like table tennis may significantly alleviate anxiety symptoms in youth, particularly those with low self-esteem, by improving performance, enhancing self-efficacy and promoting resilience to stress, through a unique training process that fosters automated action in high-pressure situations.

Our findings may influence how physical education programmes are designed and implemented. Given the observed benefits of table tennis, incorporating such sports into school curricula could potentially enhance the well-being of students.

## Introduction

Youth mental health is one of the most concerning issues in today’s world. Recent epidemiological reports from the World Health Organization estimate that one in seven youth aged 10–19 worldwide suffers from mental disorders,^1^ with approximately 50% of these disorders beginning to develop by age 14. Among these, anxiety is the most prevalent mental health disorder in youth. The prevalence rate of youth experiencing anxiety symptoms is estimated to be approximately 32.4% in the United States,^2^ 21% in China,^3^ and 13% in the United Kingdom.^4^ Mental health disorders in children and youth can have long-lasting consequences on various aspects of their lives, including academic performance, social relationships and overall quality of life.^5–8^

People with anxiety may feel nervous in stressful situations, and their symptoms can persist for a short or long period of time depending on social factors and the individual.^9^ Symptoms of anxiety may explain cognitive and behavioural changes,^10,11^ which are associated with a lower quality of life and well-being, and poor social communication.^12^ It also links that unsatisfactory academic performance,^12^ and low self-esteem,^13^ and may increase the risk of physical health. Children and adolescents with anxiety are at increased risk of exhibiting maladaptive behaviours, such as violence,^14^ later life alcohol and drug use and suicide attempts.^15–17^

One potential approach to promoting anxiety is through physical activity (PA), particularly sports participation.^18–20^ Murphy et al.^19^ found that students who participated in sporting activities, both in school and out of school, exhibited lower levels of general anxiety compared to students who did not engage in any form of sports. Lange et al. ^20^ concluded that the effectiveness of sport participation as part of sustainable anxiety care shows significant and clinically meaningful efficacy, with virtually no adverse effects, thus offering a promising option for the prevention and treatment of anxiety disorders. Engaging in PA may have beneficial effects on cognition by potentially reducing anxiety through energy expenditure.^21,22^ Meanwhile, the reduction in anxiety levels through sports may also be due to the moderating effects of self-efficacy and self-esteem.^23,24^ Mastering sports skills, such as those involved in table tennis, could also be associated with reduced anxiety symptoms due to improved cognitive functions.^25^

However, the inability to quantify sports participation, such as the type of sports, individual preferences and social and physical contexts, makes it difficult to provide precise guidelines for real-life applications regarding how different individuals should engage in sports, what kind of sports they should participate in, and how they should practice them.^26^ For instance, table tennis is a sport with a wide base of enthusiasts in many countries, and compared to basketball or football, it may have a lower barrier to entry, be easier to learn and present a reduced risk of sports injuries. Research could potentially offer guidelines on how children, adolescents and young adults can effectively engage in table tennis programmes to prevent and reduce symptoms of anxiety. Currently, no studies have considered this issue.

Sports participation is just one facet of PA, and the extent to which individuals engage in other activities, such as sedentary behaviours and sleep, may also affect the link between sport and anxiety. Thus, this study intends to use a longitudinal approach to investigate the potential impact of table tennis on the anxiety symptoms of young people. It will examine how the mastery of table tennis skills, performance and engagement are related, as well as consider psychological factors like self-esteem, self-efficacy and resilience. Furthermore, it will take into account other PA-related factors, such as baseline sedentary behaviour and sleep patterns, as potential confounders.

## Methods and materials

### Participants

Participants were recruited from the Guilin University of Electronic Technology. The inclusion criteria were non-clinical undergraduate students aged over 18. The exclusion criteria were participants who reported a history of (1) diagnosed major depression or (2) anxiety disorder and (3) the students have table tennis training experience. Participants signed up for the study voluntarily and received no payment upon completion.

### Procedure

This longitudinal observation study was approved by the Research Ethics Committee of the University where the study was conducted (the code of ethics is 21TYYB25). The volunteered participants were invited to an on-campus laboratory from 24th September 2020 and screened for eligibility. Those who matched the inclusion and exclusion criteria were invited for further data collection sessions. Verbal consent was obtained prior to their enrolment. This recruitment process complies with the ethical standards of the institutional review board. Eligible participants provided their demographic data, including age, sex, weight and height (to calculate BMI). Then, participants completed a set of questionnaires, including one self-report PA measurement and four psychological factors (self-efficacy, self-esteem, resilience and anxiety). These data were later referred to as wave 1.

After completing these baseline measurements, the students engaged in the following non-mandatory activities in the next 8 months until 24th June 2021. The students attended physical education (PE) once a week. The PE delivered non-mandatory table tennis training sessions twice a week. Each session lasted 90 min, during which table tennis skills were taught and trained by a coach from the University. The course includes warm-up running exercises, as well as instruction in table tennis skills (forehand and backhand) and table tennis matches.

After 8 months, participants returned to the lab, completed assessments for the PA level, sedentary behaviour, sleep, undertook the table tennis assessment, self-efficacy, self-esteem, resilience and anxiety.

### Assessment material

#### PA level, sedentary behaviour and sleep

PA, sedentary behaviour and sleep patterns were assessed using the International Physical Activity Questionnaire (IPAQ). Participants reported the duration of their habitual or past-week activities.^27^ The Chinese version of IPAQ had good test–retest reliability for grouped activities, with intra-class correlation coefficients ranging from 0·74 to 0·97 for vigorous, moderate, walking and total PA, with between-test effect sizes that were small (<0.49).^28^ This study specifically concentrates on assessing the duration of PA in daily life.^29^ For analytical purposes, the study categorised PA into four distinct groups based on the PA duration obtained from the IPAQ results: ‘low PA = 0’ (engaging in 0–1 h of PA per week) served as the reference group, ‘moderate PA = 1’ (1–2 h per week), ‘moderate to high PA’ (2–3 h per week) and ‘high PA’ (four or more hour per week).^30^ Additionally, participants were also advised to omit participation in organised sports, such as table tennis classes or basketball matches, from their PA calculations.

The IPAQ subscale assessed sedentary hours through the following question: ‘In the past 7 days, how many hours per day did you typically spend sitting on weekdays’ Responses were recorded as open-text entries in terms of hours per day. Similarly, participants were asked about their sleep habits: In the past 7 days, how many hours per day did you typically spend sleep on weekdays’ Again, responses were recorded as open-text entries in terms of hours per day.

#### Table tennis assessment

The assessment for the table tennis course adhered to the guidelines set forth by Guilin University of Electronic Technology’s Table Tennis Class. These guidelines were tailored from the Chinese Table Tennis Association’s China Ping Pong Nine-Level System. The overall evaluation of the final table tennis performance encompassed three main components: master table tennis skill assessment and participation in imitation matches. The master table tennis skill scoring consisted of (1) the number of successful forehand strikes completed within 1 min, and (2) participation in an imitation match game. Students achieved a total score of 80 points if they completed 28 forehand strikes in a minute, with a two-point deduction for each strike below 28. And the standardisation of the same actions accounts for 20% of the score. The criteria for successful forehand strikes included: (1) completing the strike using a forehand manoeuvre, (2) landing the ball in the opponent’s half of the table on the forehand side, with the centre-line as the boundary, (3) completing the strikes in consecutive rounds without breaks and (4) unsuccessful strikes caused by the examiner were not counted. Each student had three opportunities, and the performance was recorded from the highest-scoring round. Finally, students participated in imitation matches. They were randomly allocated into four groups and matched against each other in 10 rounds. Points were awarded based on the number of victories, with each win adding 2 points and a maximum score of 20 points. After conversion, the maximum score for the final table tennis performance is 85 points, which combines scoring for forehand strikes and imitation matches.

#### Self-efficacy

Participants’ self-efficacy was measured with the General Self-Efficacy Scale (GSES),^31,32^ which is a 10-item self-report questionnaire reported based on a four-point Likert^33^ scale from ‘Strongly disagree’ to ‘Strongly agree’ addressing self-efficacy. The GSES generally processes acceptable reliability, with Cronbach’s Alpha ranging from 0.76 to 0.90.^32^ The scale demonstrates good internal consistency, with Alpha = 0.91 in Chinese version.^34^

#### Self-esteem

Participants’ self-esteem was measured with the Rosenberg Self-Esteem Scale (RSES),^35^ which is a 10-item self-report questionnaire reported based on a four-point Likert^33^ scale from ‘Strongly disagree’ to ‘Strongly agree’. The RSES scale possesses good internal consistency, with Cronbach’s α greater than 0.99.^36^ The Chinese version of the RSES shows good internal consistency and construct validity in Chinese young people with Cronbach’s α = 0.91.^37^

#### Resilience

Participants’ resilience was measured with the Connor–Davidson Resilience Scale (CD-RISC-25),^38^ which is a 25-item self-report questionnaire reported on a five-point scale scoring from 0 to 4, labelled as ‘Never’ to ‘Always’. The CS-RISC-25 scale possesses good internal consistency, with Cronbach’s α ranging from 0.65 to 0.91.^39^ The Chinese version of CD-RISC demonstrates good reliability and validity among Chinese adolescents, with a Cronbach’s α coefficient of 0.89.^40^

#### Anxiety

Participants’ anxiety symptoms were measured with the Hamilton Rating Scale for Anxiety (HAM-A),^41^ which is a 14-item self-report questionnaire reported on a five-point scale, labelled as ‘Not present’ to ‘Very severe’. The HAM-A processes good reliability among youth, with Cronbach’s α at 0.86.^42^ The Chinese version of the HAM-A demonstrates good internal consistency, with a Cronbach’s α of 0.77.^43^

#### Statistical analysis

The data analysis was conducted with SPSS v28 (IBM Corp., Armonk, NY), and only data of participants who responded to both wave 1 and wave 2 would be included in the analysis. First, participants’ demographic factors (age and BMI), PA levels and psychological factors (self-efficacy, self-esteem, resilience and anxiety) at baseline would be matched between sex to examine baseline differences. Repeated-measure analysis of variance (RM-ANOVA)was conducted to test the mean change in the psychological factors between wave 1 from baseline and wave 2 after 8 months controlling for covariates (sex). After which, the demographic factors, PA level, psychological factors and other PA factors (table tennis performance, sedentary hours and sleep hours) would be entered into exploratory Pearson bivariate correlations to investigate the underlying mechanisms of the influence on anxiety. Since we would conduct multiple correlation tests, we would conduct Holm–Bonferroni correction to control the family-wise error rate.^44^ Then, the psychological and PA factors were entered into a multiple Linear regression analysis, controlling for covariates (age, sex, BMI and baseline psychological factors ratings) to investigate potential predictors for the mean change in anxiety. Finally, simple slope analyses were conducted to investigate the underlying mechanisms that influenced the relationships that predicted change in anxiety.

## Result

### Sample characteristics

Initially, 304 university students were recruited, and 43 of them were excluded based on exclusion criteria, leaving 264 students for baseline measurements. Of these, 101 participants dropped out, and 163 of them completed the second wave of measurements, providing 163 available responses to be analysed. Participants’ demographic and psychological factors, including age, BMI, self-reported exercise hours, self-esteem, self-efficacy, resilience and anxiety, were matched according to sex. At baseline, more male students (72.3%) were recruited than females (27.7%). No other difference between the sexes was found. The demographic information and other details are presented in Table 1. Further analysis on the anxiety levels between those who remained in the study (*N* = 163; Mean = 22.53; SD = 6.96) and those who dropped out (*N* = 101; Mean = 23.01; SD = 7.26) controlling for sex suggested no significant difference in anxiety levels between the groups, *F*(1, 260) = 0.00, *p* = 0.956, or interaction between group and sex, *F*(1, 260) = 0.98, *p* = 0.324, and sex was not a significant covariate in this analysis, *F*(1, 260) = 1.23, *p* = 0.269. These findings suggest that the dropout did not introduce bias related to anxiety levels across the groups. When sex was not controlled for in the analysis, the results remained consistent, showing no significant difference, *F*(1, 262) = 0.290, *p* = 0.591.

**Table 1.** Sample characteristics.

Wave 1 at baseline	Male					Female								Overall				
	*N*	Mean	SD	Range		*N*	Mean	SD	Range		*F*	*p*	*η*_p_^2^	*N*	Mean	SD	Range	
Age	190	19.08	0.87	17	22	72	18.93	0.88	18	22	1.44	0.218	0.006	262	19.04	0.87	17	22
BMI	191	21.27	3.91	14.73	38.88	73	20.98	3.90	16.25	33.00	0.28	0.600	0.001	264	21.19	3.90	14.73	38.88
Self-efficacy	191	26.53	4.39	16	39	73	25.07	4.32	15	35	5.93	0.016	0.021	264	26.13	4.41	15	39
Self-esteem	191	28.27	4.66	17	40	73	27.81	4.47	18	39	0.54	0.465	0.001	264	28.14	4.60	17	40
Resilience	191	83.32	11.32	49	117	73	79.03	10.31	57	109	7.97	0.005	0.026	264	82.13	11.19	49	117
Anxiety	191	22.57	7.64	14	56	73	24.27	7.55	14	44	2.64	0.105	0.008	264	23.04	7.64	14	56
		Proportion					Proportion				*x* ^2^	*p*	*w*		Proportion			
PA levels	191	100%				73	100%				5.42	0.066	0.143	264	100%			
Inactive	173	90.6%				72	98.6%							245	92.8%			
Insufficiently active	10	5.2%				0	0.0%							10	3.8%			
Active	8	4.2%				1	1.4%							9	3.4%			
Wave 2 at 8 months	*N*	Mean	SD	Range		*N*	Mean	SD	Range		*F*	*p*	*η*_p_^2^	*N*	Mean	SD	Range	
Age	133	19.03	0.90	17	22	30	18.8	0.76	18	20	1.66	0.198	0.010	163	18.99	0.882	17	22
BMI	133	20.89	3.40	16.00	38.88	30	20.67	3.42	17.22	31.00	0.05	0.751	0.000	163	20.85	3.39	16.00	38.88
Self-efficacy	133	27.68	4.49	17	40	30	25.57	4.25	18	37	5.43	0.020	0.033	163	27.29	4.509	17	40
Self-esteem	133	28.92	4.00	17	40	30	28.63	3.85	18	40	0.07	0.724	0.000	163	28.87	3.964	17	40
Resilience	133	81.76	15.23	0	117	30	81.47	12.89	52	118	0.00	0.922	0.000	163	81.71	14.793	0	118
Anxiety	133	22.86	8.48	0	49	30	25.5	9.69	14	46	2.34	0.136	0.014	163	23.35	8.743	0	49
		Proportion					Proportion				*x* ^2^	*p*	*w*		Proportion			
PA levels	133	100%				30	100%				3.59	0.166	0.148	163	100%			
Inactive	59	44.4%				19	63.3%							78	47.9%			
insufficiently active	69	51.9%				10	33.3%							79	48.5%			
Active	5	3.8%				1	3.3%							6	6.0%			
											*F*	*p*	*η*_p_^2^					
Table tennis performance	133	34.67	4.70	0.00	40.00	30	35.73	2.86	28.00	40.00	1.48	0.236	0.009	163	34.87	4.43	0	40
Sedentary hours (week)	133	9.72	6.27	0.00	25.00	30	10.19	6.65	0.00	28.00	0.19	0.712	0.001	163	9.81	6.32	0	28
Sleeping hours (day)	133	6.42	2.71	0.00	10.00	30	6.47	3.39	0.00	10.00	0.01	0.937	0.000	163	6.43	2.84	0	10

### Longitudinal changes over 8 months

Self-efficacy, self-esteem, resilience and anxiety were entered into a repeated-measures ANOVA, with sex as the covariate, to estimate the mean change over the 8-month period.

### Change in self-efficacy

**Figure 1.**
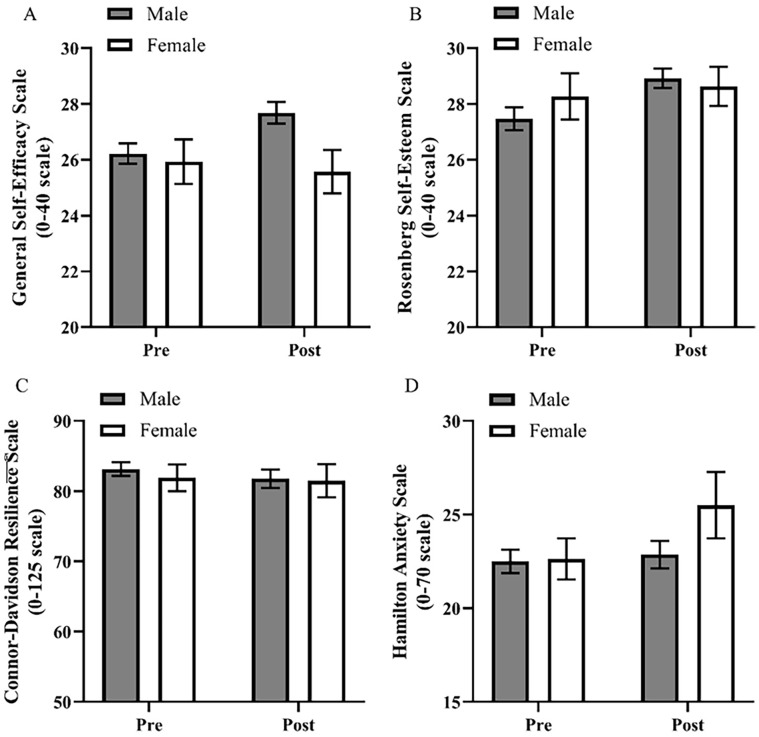
Psychology longitudinal change over 8 months.

Self-efficacy significantly increased over the 8-month period, *F*(1, 161) = 11.12, *p* = 0.001, *η*_p_^2^ = 0.065, as shown in Figure 1A. Sex was not a significant covariate, *F*(1, 161) = 2.81, *p* = 0.096, *η*_p_^2^ = 0.017, and there was no interaction between the mean change of self-efficacy and sex, *F*(1, 161) = 3.20, *p* = 0.076, *η*_p_^2^ = 0.019. This suggests a significant increase in self-efficacy.

### Change in self-esteem

Self-esteem significantly increased over the 8-month period, *F*(1, 161) = 11.79, *p* < 0.001, *η*_p_^2^ = 0.068, as shown in Figure 1B. Sex was not a significant covariate, *F*(1, 161) = 0.13, *p* = 0.722, *η*_p_^2^ = 0.001, and there was no interaction between the mean change of self-esteem and sex, *F*(1, 161) = 1.21, *p* = 0.273, *η*_p_^2^ = 0.007. This suggests that there was no significant change in self-esteem.

### Change in resilience

Resilience showed no significant change over the 8-month period, *F*(1, 161) = 1.45, *p* = 0.231, *η*_p_^2^ = 0.009, as shown in Figure 1C. Sex was not a significant covariate, *F*(1, 161) = 0.11, *p* = 0.738, *η*_p_^2^ = 0.001, and there was no interaction between the mean change of resilience and sex, *F*(1, 161) = 0.13, *p* = 0.724, *η*_p_^2^ = 0.001. This suggests that resilience did not change significantly for either male or female students.

### Change in anxiety

As shown in Figure 1D, anxiety demonstrated no significant change over the 8-month period, *F*(1, 161) = 0.31, *p* = 0.576, *η*_p_^2^ = 0.002. Sex was not a significant covariate, *F*(1, 161) = 2.78, *p* = 0.097, *η*_p_^2^ = 0.017, and there was no interaction between the mean change of anxiety and sex, *F*(1, 161) = 0.97, *p* = 0.327, *η*_p_^2^ = 0.006. This indicates that anxiety did not significantly change in either male or female students.

### Exploratory correlation

Participants’ demographic factors (age and BMI), psychological factors (self-efficacy, self-esteem, resilience and anxiety) with respect to Waves 1 and 2 and their mean changes and PA factors (PA level, table tennis test performance, sedentary and sleep) were analysed using Pearson correlations to further explore their potential underlying roles. The correlation matrix is shown in Table 2. Since we are most interested in anxiety, as shown in Table 2, the mean change of anxiety over the 8-month period was only correlated with the psychological factors, including self-efficacy at wave 2 (*r* = −0.26, *p* < 0.001), self-efficacy mean change over the 8 months (*r* = −0.30, *p* < 0.001), self-esteem at wave 2 (*r* = −0.22, *p* = 0.003), self-esteem mean change over the 8 months (*r* = −0.23, *p* = 0.004), resilience at wave 2 (*r* = −0.17, *p* = 0.031), resilience mean change over the 8 months (*r* = −0.17, *p* = 0.028) and anxiety itself at wave 1 and wave 2. It was not directly correlated with PA factors.

**Table 2.** Correlation matrix.

Variables	1. Age	2	3	4	5	6	7	8	9	10	11	12	13	14	15	16	17	18
2. BMI	rs = −0.035 ps = 0.655																	
3. Self-efficacy wave 1	rs = −0.054 ps = 0.496	rs = −0.02 ps = 0.802																
4. Self-efficacy wave 2	rs = −0.184 ps = 0.019	rs = −0.04 ps = 0.615	rs = 0.323 ps < 0.001															
5. ΔSelf-efficacy	rs = −0.118 ps = 0.135	rs = −0.019 ps = 0.814	rs = −0.549 ps < 0.001	rs = 0.614 ps < 0.001														
6. Self-esteem wave 1	rs = −0.243 ps = 0.002	rs = 0.019 ps = 0.808	rs = 0.33 ps < 0.001	rs = 0.178 ps = 0.023	rs = −0.118 ps = 0.132													
7. Self-esteem wave 2	rs = −0.198 ps = 0.011	rs = 0.033 ps = 0.673	rs = 0.28 ps < 0.001	rs = 0.577 ps < 0.001	rs = 0.276 ps < 0.001	rs = 0.37 ps < 0.001												
8. ΔSelf-esteem	rs = 0.071 ps = 0.368	rs = 0.009 ps = 0.913	rs = −0.088 ps = 0.263	rs = 0.299 ps < 0.001	rs = 0.338 ps < 0.001	rs = −0.656 ps < 0.001	rs = 0.458 ps < 0.001											
9. Resilience wave 1	rs = −0.06 ps = 0.447	rs = −0.005 ps = 0.951	rs = 0.629 ps < 0.001	rs = 0.379 ps < 0.001	rs = −0.19 ps = 0.015	rs = 0.508 ps < 0.001	rs = 0.442 ps < 0.001	rs = −0.126 ps = 0.108										
10. Resilience wave 2	rs = −0.067 ps = 0.395	rs = 0.055 ps = 0.488	rs = 0.269 ps < 0.001	rs = 0.581 ps < 0.001	rs = 0.288 ps < 0.001	rs = 0.288 ps < 0.001	rs = 0.65 ps < 0.001	rs = 0.252 ps = 0.001	rs = 0.512 ps < 0.001									
11. ΔResilience	rs = −0.025 ps = 0.749	rs = 0.066 ps = 0.406	rs = −0.222 ps = 0.004	rs = 0.336 ps < 0.001	rs = 0.482 ps < 0.001	rs = −0.1 ps = 0.205	rs = 0.361 ps < 0.001	rs = 0.389 ps < 0.001	rs = −0.26 ps < 0.001	rs = 0.696 ps < 0.001								
12. Anxiety wave 1	rs = 0.17 ps = 0.03	rs = −0.005 ps = 0.947	rs = −0.189 ps = 0.016	rs = −0.162 ps = 0.038	rs = 0.014 ps = 0.857	rs = −0.236 ps = 0.002	rs = −0.284 ps < 0.001	rs = −0.005 ps = 0.949	rs = −0.307 ps < 0.001	rs = −0.194 ps = 0.013	rs = 0.038 ps = 0.629							
13. Anxiety wave 2	rs = 0.169 ps = 0.031	rs = 0.071 ps = 0.371	rs = −0.079 ps = 0.315	rs = −0.355 ps < 0.001	rs = −0.247 ps = 0.001	rs = −0.138 ps = 0.078	rs = −0.411 ps < 0.001	rs = −0.202 ps = 0.01	rs = −0.263 ps < 0.001	rs = −0.299 ps < 0.001	rs = −0.117 ps = 0.137	rs = 0.567 ps < 0.001						
14. ΔAnxiety	rs = 0.04 ps = 0.613	rs = 0.087 ps = 0.267	rs = 0.084 ps = 0.289	rs = −0.264 ps < 0.001	rs = −0.303 ps < 0.001	rs = 0.058 ps = 0.463	rs = −0.217 ps = 0.005	rs = −0.232 ps = 0.003	rs = −0.021 ps = 0.788	rs = −0.169 ps = 0.031	rs = −0.172 ps = 0.028	rs = −0.268 ps < 0.001	rs = 0.642 ps < 0.001					
15. PA hours wave 1	rs = 0.163 ps = 0.038	rs = 0.01 ps = 0.901	rs = 0.103 ps = 0.192	rs = 0.087 ps = 0.271	rs = −0.009 ps = 0.909	rs = −0.042 ps = 0.597	rs = −0.021 ps = 0.794	rs = 0.023 ps = 0.769	rs = 0.161 ps = 0.04	rs = 0.036 ps = 0.644	rs = −0.093 ps = 0.236	rs = −0.022 ps = .0781	rs = 0.02 ps = 0.798	rs = 0.044 ps = 0.576				
16. PA hours wave 2	rs = .0162 ps = .0039	rs = 0.053 ps = 0.501	rs = 0.069 ps = 0.383	rs = 0.075 ps = 0.34	rs = 0.009 ps = 0.908	rs = −0.03 ps = 0.705	rs = −0.06 ps = 0.45	rs = −0.02 ps = 0.802	rs = 0.168 ps = 0.033	rs = 0.001 ps = 0.987	rs = −0.138 ps = 0.078	rs = −0.019 ps = 0.812	rs = 0.031 ps = 0.691	rs = 0.054 ps = 0.493	rs = 0.943 ps < 0.001			
17. Table tennis performance	rs = −0.094 ps = 0.235	rs = 0.076 ps = 0.334	rs = 0.100 ps = 0.206	rs = 0.075 ps = 0.34	rs = −0.017 ps = 0.833	rs = 0.024 ps = 0.758	rs = 0.086 ps = 0.272	rs = 0.047 ps = 0.551	rs = 0.107 ps = 0.173	rs = 0.103 ps = 0.193	rs = 0.026 ps = 0.745	rs = −0.011 ps = 0.888	rs = −0.069 ps = 0.382	rs = −0.07 ps = 0.373	rs = −0.005 ps = 0.946	rs = −0.029 ps = 0.716		
18. Sedentary hours	rs = −0.044 ps = 0.575	rs = 0.042 ps = 0.596	rs = −0.118 ps = 0.134	rs = −0.212 ps = 0.007	rs = −0.089 ps = 0.259	rs = −0.144 ps = 0.067	rs = −0.135 ps = 0.087	rs = 0.028 ps = 0.72	rs = −0.069 ps = 0.381	rs = −0.081 ps = 0.302	rs = −0.034 ps = 0.67	rs = 0.013 ps = 0.871	rs = −0.065 ps = 0.407	rs = −0.088 ps = 0.262	rs = 0.015 ps = 0.849	rs = 0.025 ps = 0.748	rs = 0.141 ps = 0.072	
19. Sleep	rs = −0.007 ps = 0.934	rs = 0.041 ps = 0.607	rs = −0.145 ps = 0.065	rs = −0.104 ps = 0.186	rs = 0.029 ps = 0.714	rs = −0.009 ps = 0.909	rs = 0.009 ps = 0.909	rs = 0.016 ps = 0.839	rs = −0.035 ps = 0.66	rs = 0.128 ps = 0.105	rs = 0.172 ps = 0.028	rs = −0.136 ps = 0.083	rs = −0.123 ps = 0.117	rs = −0.017 ps = 0.825	rs = 0.057 ps = 0.471	rs = 0.04 ps = 0.613	rs = 0.058 ps = 0.461	rs = 0.337 ps < 0.001
Sample size	163	163	163	163	163	163	163	163	163	163	163	163	163	163	163	163	163	163

However, after adjusting for multiple comparisons using the Holm–Bonferroni correction to control for family-wise error, the mean change in anxiety over the 8-month period was found to be significantly correlated with self-efficacy at wave 2 (*p*_adj_ < 0.001), the mean change in self-efficacy over the 8 months (*p*_adj_ = 0.003), self-esteem at wave 2 (*p*_adj_ = 0.012) and the mean change in self-esteem over the 8 months (*p*_adj_ = 0.024). In contrast, we failed to reject the null hypothesis, indicating no correlation between the mean change in anxiety and resilience at wave 2 (*p*_adj_ = 0.51) or the mean change in resilience over the 8 months (*p*_adj_ = 0.55). As a result, self-esteem and self-efficacy will be the focus of our subsequent analysis.

### Linear regression model predicting anxiety

The psychological factors (change in self-efficacy, self-esteem and resilience) and PA factors (table tennis scores, sedentary hours and sleep hours) were entered into multiple regression, controlling for covariates (age, sex, BMI, baseline self-efficacy, self-esteem, resilience at wave 1, and PA level at wave 1). The adjusted results suggested that change in self-efficacy (*β* = −0.43, *t* = −2.43, *p* = 0.016), change in self-esteem (*β* = −0.38, *t* = −2.46, *p* = 0.015) and sedentary hours (*β* = −0.17, *t* = −1.99, *p* = 0.049) were significant predictors of change in anxiety, explaining most of the variance (*R*^2^ = 0.51, *F*(11, 151) = 4.93, *p* < 0.001).

Moreover, the adjusted multiple regression model suggested that change in anxiety was predicted by sedentary hours (*β* = −0.45, *t* = −3.15, *p* = 0.003) and table tennis performance (*β* = −0.42, *t* = −2.38, *p* = 0.021) among those who reported decreased self-esteem, whereas these variables did not predict change in anxiety among those who reported increased self-esteem. This suggests that the change in self-esteem was potentially moderating these slopes. The adjusted multiple regression model also suggested that change in anxiety was predicted by self-esteem (*β* = −0.85, *t* = −2.64, *p* = 0.011) among those who reported decreased self-efficacy, whereas sedentary hours (*β* = −0.30, *t* = −2.33, *p* = 0.023) and table tennis performance (*β* = −0.24, *t* = −2.12, *p* = 0.037) predicted change in anxiety among those who reported decreased self-efficacy, indicating that self-efficacy might also moderate these slopes as well.

### Linear regression model predicting self-esteem and self-efficacy

Since we found that the change in self-esteem and self-efficacy were associated with the change in anxiety, and both self-esteem and self-efficacy significantly increased over the 8 months, we would further test their relationship with PA factors (sedentary behaviour, table tennis performance and sleep). They were entered into a multiple regression model controlling for covariates (age, sex, BMI, baseline self-efficacy, self-esteem, resilience at wave 1, and PA level at wave 1). As shown in Table 3, the change in self-esteem was only predicted by the controlled covariates, but not sedentary behaviour (*p* = 0.486), table tennis performance (*p* = 0.517) or sleep (*p* = 0.939). Similarly, the change in self-efficacy was only predicted by the controlled covariates, but not sedentary behaviour (*p* = 0.237), table tennis performance (*p* = 0.659) or sleep (*p* = 0.281). These results suggested that our measured PA factors (sedentary behaviour, table tennis performance and sleep) were not directly associated with the change in self-esteem or self-efficacy.

**Table 3.** Multiple regression analysis controlling for covariates.

Dependent	Predictor	*B*	SE	*t*	*p*	Tolerance	VIF
ΔAnxiety	ΔSelf-efficacy	−0.43	0.18	−2.43	0.016	0.363	2.755
	ΔSelf-esteem	−0.38	0.15	−2.46	0.015	0.438	2.283
	ΔSedentary	−0.17	0.09	−1.99	0.049	0.916	1.092
	Covariates	*B*	SE	*t*	*p*	Tolerance	VIF
	Age	0.42	0.65	0.65	0.516	0.856	1.169
	BMI	0.20	0.16	1.27	0.205	0.990	1.011
	Sex	1.90	1.41	1.35	0.180	0.928	1.078
	Self-efficacy at wave 1	0.01	0.21	0.07	0.946	0.360	2.782
	Self-esteem at wave 1	−0.27	0.21	−1.29	0.198	0.301	3.318
	Resilience at wave 1	−0.09	0.07	−1.20	0.231	0.433	2.309
	Anxiety at wave 1	−0.38	0.08	−4.62	<.001	0.860	1.163
	PA at wave 1	0.59	0.74	0.79	0.431	0.913	1.095
	Predictor	*B*	SE	*T*	*p*	Tolerance	VIF
ΔSelf-esteem	Table tennis performance	0.03	0.09	0.54	0.589	0.960	1.042
	Sedentary	−0.08	0.06	−1.38	0.168	0.960	1.042
	Sleep	0.01	0.14	0.09	0.931	0.944	1.059
	Covariates	*B*	SE	*T*	*p*	Tolerance	VIF
	Age	−0.52	0.33	−1.58	0.117	0.893	1.12
	BMI	0.03	0.08	0.39	0.698	0.997	1.003
	Sex	−0.42	0.72	−.58	0.562	0.969	1.032
	Self-efficacy at wave 1	0.00	0.08	0.00	1.000	0.604	1.655
	Self-esteem at wave 1	−0.88	0.07	−12.34	<0.001	0.683	1.464
	Resilience at wave 1	0.12	0.04	3.26	0.001	0.468	2.138
	Anxiety at wave 1	−0.07	0.04	−1.76	0.081	0.879	1.138
	PA at wave 1	−0.31	0.39	−0.80	0.426	0.923	1.084

### Moderating the relationship between self-efficacy, self-esteem and anxiety

**Figure 2.**
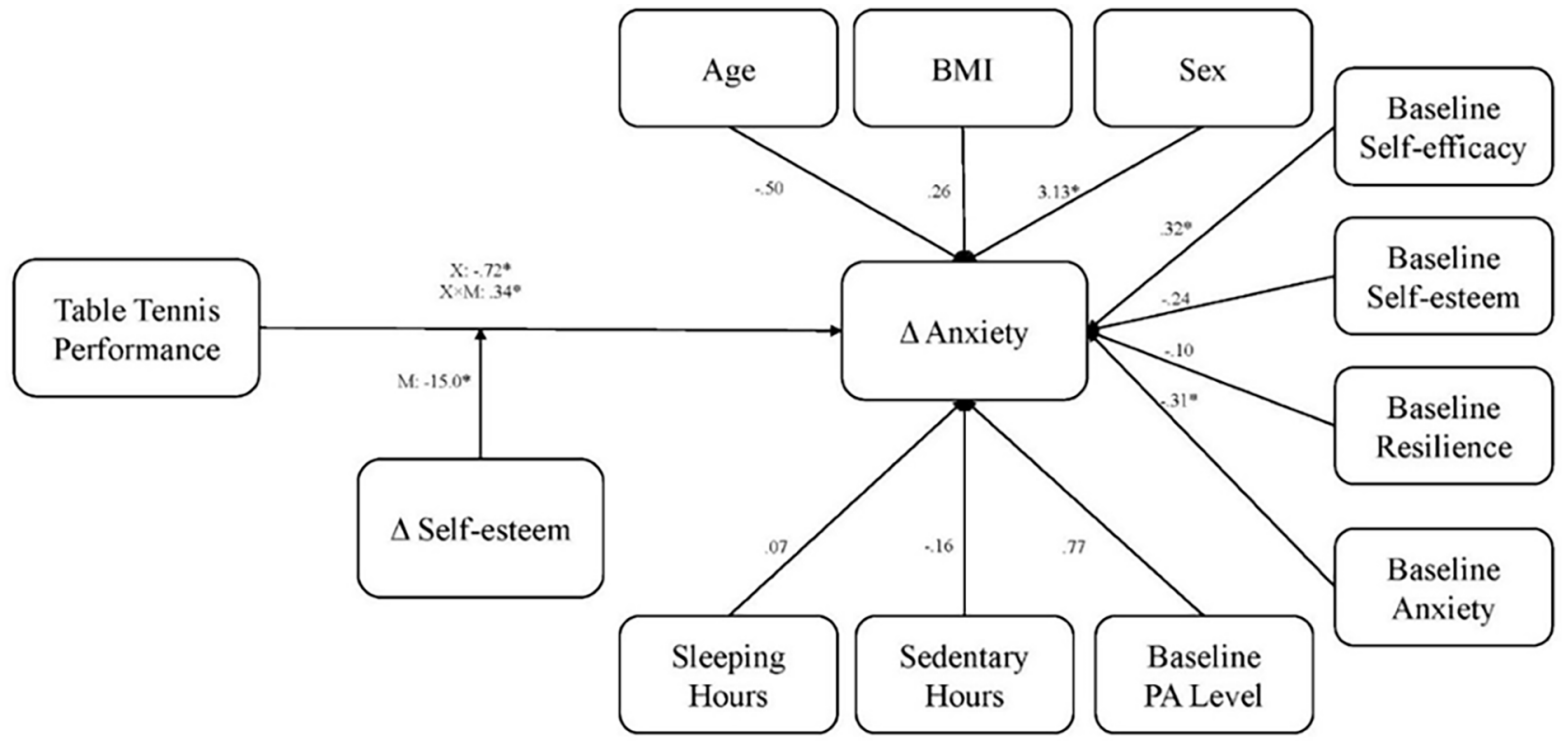
Full moderation model.

Simple slope analyses indicated that the models of sedentary behaviour moderation were insignificant, as models were moderating the effect on change in anxiety with self-efficacy. The effect of table tennis performance on change in anxiety was significantly moderated by the change in self-esteem (*R*^2^ = 0.54, *F*(13, 149) = 5.16, *p* < 0.001). As demonstrated in Figure 2, simple slope analysis controlling for characteristic factors (age, BMI and sex), Baseline psychological factors (self-efficacy, self-esteem, resilience and anxiety) and PA factors (PA level at wave 1, sedentary behaviour and sleep) suggested that the effect of table tennis on the change in anxiety was moderating the effect of self-esteem on anxiety, where sex (*p* = 0.022), baseline self-efficacy (*p* = 0.044) and baseline anxiety (*p* < 0.001) were the only significant covariates. There was a significant two-way interaction between self-esteem and table tennis (*F*(1, 149) = 7.30, *p* = 0.008). Table 4 shows no significant multicollinearity among the covariates.

**Table 4.** Simple slop analysis of self-esteem moderating effect of table tennis.

Model items	*β*	SE	*t*	*p*		
Table tennis performance	−0.72	0.25	−2.83	0.005		
ΔSelf-esteem	−15.02	4.49	−3.35	0.001		
ΔSelf-esteem × Table tennis performance	0.34	0.13	2.70	0.008		
Covariates	*β*	SE	*t*	*p*	Tolerance	VIF
Age	0.50	0.63	0.80	0.428	0.860	1.163
BMI	0.26	0.15	1.68	0.095	0.988	1.012
Sex	3.13	1.35	2.31	0.022	0.956	1.046
Self-efficacy at wave 1	0.32	0.16	2.03	0.044	0.582	1.72
Self-esteem at wave 1	−0.24	0.16	−1.57	0.118	0.330	3.028
Resilience at wave 1	−0.10	0.07	−1.49	0.139	0.432	2.314
Anxiety at wave 1	−0.31	0.08	−3.93	0.000	0.836	1.196
PA at wave 1	0.77	0.72	1.06	0.289	0.912	1.097
Sedentary	−0.16	0.09	−1.78	0.076	0.825	1.212
Sleep	0.07	0.20	0.36	0.717	0.832	1.202

**Figure 3.**
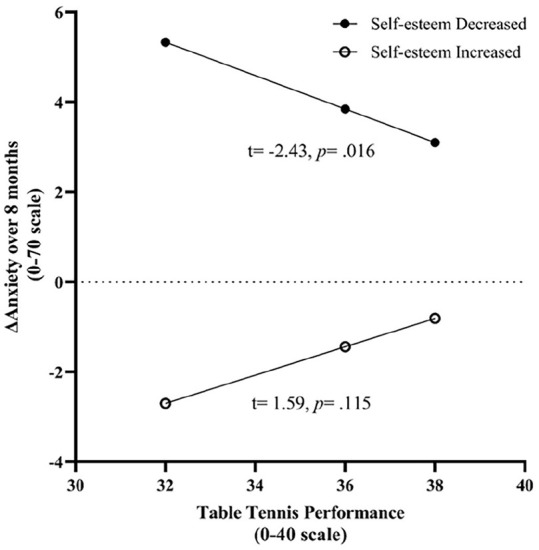
Moderation model for self-esteem and table tennis performance.

Specifically, as shown in Figure 3, the development in anxiety symptom ratings was only related to table tennis performance when self-esteem decreased (*t* = −2.43, SE = 0.15, *p* = 0.016), whereas there was no relationship between table tennis performance and change in anxiety symptom ratings when self-esteem increased (*t* = 1.59, SE = 0.31, *p* = 0.115). This moderation model suggested that when students reported increased anxiety associated with their decrease in self-esteem, those with high table tennis performance seemed significantly buffered the development of anxiety.

## Discussion

The current study examined the longitudinal impact of table tennis participation and psychological factors (self-efficacy, self-esteem and resilience) on anxiety symptoms among 163 university youth (81.6% male). This is the first study to investigate the role of regular long-term table tennis exercises on youth’ anxiety symptoms. Overall, self-esteem and self-efficacy levels increased during the study period, but these increases were not associated with the PA factors. The results indicated that changes in self-efficacy, self-esteem and sedentary hours were negatively related to changes in anxiety symptoms, with an increase in self-efficacy, self-esteem, or sedentary hours predicting a decrease in anxiety symptoms. Further analysis revealed an exploratory moderation model that the change in self-esteem moderated the relationship between students’ table tennis performance and anxiety symptoms. Specifically, when students reported increased anxiety associated with decreased self-esteem, those with high table tennis performance seemed significantly buffered the development of anxiety.

### Longitudinal change in 8 months

The current study first reported no change in anxiety over 8 months of observation. Relevant longitudinal studies reporting the effect of sports on anxiety over time were very limited. As reviewed by Eime et al.^45^ and Dale et al.,^46^ only two longitudinal studies investigated relevant topics.^47,48^ The current results are partially consistent with the findings of these previous longitudinal studies. In Dimech and Seiler^47^ study, 208 primary school children aged 7–8 were interviewed in a structured manner. Their parents and teachers rated the children’s symptoms of social anxiety, behaviour and sports outside of school at the beginning of the study and 1 year later. Their results failed to find a significant general effect of time on anxiety, whereas children who engaged in over 2 h of sports per week showed a decrease in self-reported social anxiety symptoms over the year. Similarly, Findlay and Coplan^48^ collected information from 201 10-year-old children about their psychological factors and sports at the beginning and 1 year later, but they failed to find a significant direct effect of time on anxiety either, whereas children who were initially shy and engaged in sports activities for a year exhibited a decline in their anxiety symptoms over time. It makes sense for these results, including ours, did not find a significant direct effect of time on anxiety because longitudinal design studies aimed to observe anxiety among teenagers, where we did not deliver sports as an intervention to compare with a control group. During the 8 months, our students participated in weekly table tennis training, and thus we would not expect a significant change.

### Predicting the longitudinal change

The current study reported no evidence to support that table tennis performance, sedentary behaviour, or sleep hours predicted changes in self-esteem or self-efficacy. Generally, sports intervention increases self-esteem and self-efficacy, as reviewed in previous quantitative analyses.^46,49^ However, the effect may differ between different types of sports intervention. Dimech and Seiler^47^ suggested that individual sports are weakly associated with self-esteem. Instead, team PA involving a strong social component potentially has a greater impact on these psychological variables. Babiss and Gangwisch^50^ investigated the effect of different types of sports on psychological variables among youth with a cross-sectional design analysis to examine the effects of various sports, including team sports and individual exercises, on self-esteem and depressive symptoms. The results suggested that participation in team sports was associated with higher self-esteem and lower levels of depressive symptoms compared to individual exercises. Similarly, Eime et al.^45^ explored the relationship between sport participation and psychological well-being among Australian youth and adults in a cross-sectional design study to examine how different types of sports (team and individual) influenced participants’ psychological well-being. The results suggested that those who participated in team sports exhibited higher levels of psychological well-being than those who participated in individual or no sports. In the current study, table tennis was an individual sport which contained a weak social component to improve self-esteem or self-efficacy. The improvement of self-esteem and self-efficacy might not result from table tennis performance but rather from other potential factors not measured in the current study.

One of the potential explanations is that the competitive matches of table tennis were delivered during the PE and the final performance assessment. Consequently, the sense of achievement acquired from the competitive matches could potentially benefit other sense of agency factors and eventually moderate the development of anxiety symptoms. For example, team sports achievement is associated with team sports achievement and self-esteem, and individual sports may process the same association but are weaker than team sports, whereas, on the other hand, violation of the expected achievement may lead to the reduction in agency factors.^51^ Therefore, students who won competition matches and scored highly in table tennis performance may experience a higher sense of achievement and potentially higher self-esteem. However, this might not always be the case, as winning the competition was not the most weighted item in the current table tennis assessment, accounting for only 20%, whereas the other 80% weight scored the skills in forehead strike. Many students may still score excellent performance in the forehead strike assessment, and they may not process the expected achievement in the following matches, such that we would not observe pronounced associations directly between table tennis performance and psychological factors. Future studies could consider including the sense of achievement to explore how individual sports affect the sense of agency.

Our multiple regression model controlling for covariates suggested that the change in self-efficacy, self-esteem and sedentary hours was predicting the change in anxiety. That is, an increase in self-efficacy, self-esteem or sedentary hours predicted a decrease in anxiety. The effect of self-esteem and self-efficacy on anxiety has been demonstrated throughout the literature.^35,52–54^ Self-esteem and self-efficacy enhance an individual’s confidence and control,^25,55^ enabling them to adopt effective coping strategies such as seeking social support, thus reducing their likelihood of experiencing anxiety. Additionally, high levels of self-esteem and self-efficacy contribute to a greater sense of personal worth and value, aiding in coping with stressful situations.

However, we also found that higher sedentary hours predicted reduced anxiety, contradicting some studies that reported that higher sedentary hours lead to increased anxiety,^56^ although previous results were inconsistent.^57^ One possible reason is that our students generally reported very few sedentary hours. As reported in Table 1, the average sedentary hour of our students was 9.8 ± 6.32 h/week, which was about 1.8 h/day (ranging from 0 to 4 h/day), whereas the sedentary hour reported in other studies were much higher. For example, Lee and Kim^58^ reported a significant increase in stress, anxiety and depression among university students with increased screen time, whereas the mean screen time of their students was 7.96 h/day. Cao et al.^59^ reported that high-school youth who spent more than 2 h/day in front of screens had a 36% greater chance of experiencing anxiety symptoms than those who spent less than 2 h/day. On the contrary, Griffiths et al.^60^ reported that girls who spent less than 2 h of screen entertainment were at a lower risk of anxiety symptoms. Hypothetically, in some cases, low levels of sedentary behaviour could indicate inability or unwillingness to participate in relaxing or enjoyable activities, leading to anxiety. Therefore, previous evidence only endorses the effect of high sedentary hours predicting anxiety, and the impact of low sedentary hours among youth remains unclear, requiring further clarification through future research.

### Moderating the longitudinal changes

The primary finding of the current study was an exploratory moderation model on the effect of table tennis performance on the development of anxiety symptoms after controlling for covariates. Specifically, high table tennis performance significantly buffered the development of anxiety symptoms only when anxiety symptoms were associated with decreased self-esteem. This moderation model is partially aligned with the results from previous studies, although very few studies have investigated the effect of playing table tennis on mental health. In a randomised control trial study, investigating the effects of different types of sports, including table tennis, on college students’ anxiety and depression levels effectively alleviated anxiety symptoms but not depression levels,^61^ whereas it did neither support whether table tennis was superior to the other sports nor suggest the mechanism about how the reduction of anxiety occurred. In a broader view, consistent effects have been reported on aerobic sports in reducing anxiety through quantitative review, whereas the effect is weaker than psychological treatments or medication,^62^ and the mechanism remains unclear. Regular table tennis training, under expert guidance, could be considered aerobic sports. The current exploratory moderation model could probably provide an initial explanation about why the effect of table tennis was weak in reducing anxiety. That is, the effect was moderated by self-esteem, which only served as a prevention to buffer the development of anxiety symptoms when self-esteem went down. As suggested before, students’ self-esteem plays an important role in anxiety, and it was not influenced by any sports factors measured in this study, which requires further investigation.

The current study cannot directly provide evidence to justify the buffering effect of table tennis on anxiety development. An alternative explanation for the impact of table tennis on anxiety could be related to its role as a form of regular aerobic exercise and its potential influence on heart rate variability (HRV). HRV, an index of cardiovascular function, measures the variation in time between successive heartbeats and has been linked to mental health issues, including anxiety. Higher HRV, reflecting reduced emotional regulation, is associated with increased anxiety symptoms.^63^ Regular aerobic sport is suggested to reduce HRV among anxiety patients, thereby alleviating their symptoms.^64^ The effect of table tennis on HRV has shown inconsistent results across different studies. Although Picabea et al.^65^ found no change in HRV before and after table tennis matches, Djokic and Zagatto^66^ reported lower HRV in elite table tennis athletes than amateur players. Consequently, the discrepancy might be that elite athletes undergo long-term regular practice to reach their level, whereas short-term acute matches may not yield significant changes in HRV. In the current study context, students engaged in regular table tennis exercises for 8 months. Those who put effort into achieving high scores in the final assessment may have better control over their HRV than others, even if they did not necessarily win matches or experience a sense of achievement. As a result, the buffering effect of table tennis on anxiety ratings could be observed when self-esteem decreased, potentially due to improved HRV control. Hypothetically, this effect may not be as pronounced in those who exhibited decreased anxiety ratings due to self-esteem development, which requires further investigation in future studies.

### Limitations

There are several limitations to consider in this longitudinal study. Firstly, the sample was biased towards male students (81.6% male), and many students dropped out of the study, not completing the 8-month non-mandatory PE course. This resulted in a relatively small and biased sample size for a longitudinal study despite the moderate effect sizes. Although sex was not found to be a significant covariate of change in self-efficacy and self-esteem over the 8 months, these results should be interpreted with caution regarding the imbalance of sex ratios. Consequently, the results may not be confidently generalised to all university youth and require future replication in more comprehensive samples. Secondly, most measurements, including all psychological factors, PA level, sedentary and sleep hours, except for table tennis, relied on self-report questionnaires, which may be biased because of inaccurate estimation of time^67^ and intention-behavioural gap.^68^ Ideally, objective measurements, such as accelerometers, should be used to measure PA factors. However, in practice, one must also consider whether these devices would be affordable for a longitudinal design across the schedule in future studies. Third, as with all studies, there might be potential confounding factors that we failed to consider and control, despite including some covariates. For example, we could not provide evidence to suggest the cause of the increase in self-esteem and self-efficacy over 8 months, which warrants future investigation. Lastly, it is essential to note that this study’s longitudinal design does not allow for causal inferences, as there was no control group. There was no sample size calculation during the designing stage because we aimed to include the whole cohort of university students. The study is exploratory, and the findings inform future research directions, including controlled experimental studies or larger-scale longitudinal investigations, to further explore and confirm these relationships.

### Implications and directions for future research

The present study provided insights with practical implications in multiple areas. Our findings underscore the potential of table tennis as an effective sports in mitigating anxiety symptoms, particularly when self-esteem declines. Educational institutions and sports organisations could refer to these results by developing and promoting table tennis programmes tailored to university students to protect students’ mental health. Furthermore, the study accentuates the need to prioritise self-esteem and self-efficacy in interventions designed to alleviate anxiety. As such, mental health professionals may consider incorporating strategies to boost self-esteem and self-efficacy into existing treatments, either as standalone components or in combination with sports, such as table tennis.

Future research should address the current study’s limitations, such as sample size, gender bias and reliance on self-report measures. Large-scale, controlled studies that include balanced-sex participants would provide more robust evidence regarding the relationships between table tennis, self-efficacy, self-esteem and anxiety. Additionally, implementing objective measures of sports, such as accelerometers, and physiological measures of mental health, such as HRV, can enhance the validity of future studies. Lastly, examining the potential moderating effects of other psychological factors and exploring the mechanisms underlying the interaction between sports and mental health would contribute to a more comprehensive understanding of this complex relationship.

## Conclusion

In conclusion, the current study was the first to examine the longitudinal impact of table tennis exercise and psychological factors on anxiety. We reported that the development of anxiety symptoms was only negatively predicted by the longitudinal change of self-esteem and self-efficacy but not by sports factors directly. We also suggested an exploratory moderation model, where the relationship between self-esteem and the longitudinal change in anxiety was moderated by table tennis performance. Specifically, table tennis exercise buffered the development of anxiety symptoms that were associated with decreased self-esteem. Limitations of this study included a relatively small sample size with a biased sex ratio (81.6% male), resilience on self-report measurements and the lack of control groups to establish causal relationships, which requires future studies to replicate and confirm the results. Despite the limitations, the current study provides insight to explain the mechanism of how sports, such as table tennis, interacts with mental health.

## Supplemental Material

sj-docx-1-smo-10.1177_20503121241258736 – Supplemental material for How to reduce anxiety symptoms through individual sport in youth: A longitudinal study over 8-month observation Supplemental material, sj-docx-1-smo-10.1177_20503121241258736 for How to reduce anxiety symptoms through individual sport in youth: A longitudinal study over 8-month observation by Lin Wang, Tianle Zhang, Weihao Huang, Leyuan Gou, Ming Zhong, Qiaohan Liu and Yihao Liu in SAGE Open Medicine

sj-docx-2-smo-10.1177_20503121241258736 – Supplemental material for How to reduce anxiety symptoms through individual sport in youth: A longitudinal study over 8-month observation Supplemental material, sj-docx-2-smo-10.1177_20503121241258736 for How to reduce anxiety symptoms through individual sport in youth: A longitudinal study over 8-month observation by Lin Wang, Tianle Zhang, Weihao Huang, Leyuan Gou, Ming Zhong, Qiaohan Liu and Yihao Liu in SAGE Open Medicine
