# Supplementary material for: How to reduce anxiety symptoms through individual sport in youth: A longitudinal study over 8-month observation
Source: SAGE Open Med. 2024 Jun 17;12:20503121241258736. doi: 10.1177/20503121241258736 (PMC11185022; doi:10.1177/20503121241258736)
Supplement: sj-docx-1-smo-10.1177_20503121241258736 – Supplemental material for How to reduce anxiety symptoms through individual sport in youth: A longitudinal study over 8-month observation [file sj-docx-1-smo-10.1177_20503121241258736.docx]

Self-Efficacy Scale Questionnaire (SEF)

Name: _______ Gender: _______ Age: _______ Major: _______ Job position: _______

College entrance exam score: _______ Email: ________________ Phone: ________________

Previous year's exercise habits (weekly exercise time, frequency, type):

Instructions: The following survey is about your general perception of yourself on a regular basis. Please answer according to your actual situation (real feelings). There is no right or wrong answer, and you don't need to overthink each statement.

1. I can always manage to solve difficult problems if I try hard enough.

a. Strongly agree b. Agree

c. Disagree d. Strongly disagree

2. If someone opposes me, I can find means and ways to get what I want.

a. Strongly agree b. Agree

c. Disagree d. Strongly disagree

3. It is easy for me to stick to my aims and accomplish my goals.

a. Strongly agree b. Agree

c. Disagree d. Strongly disagree

4. I am confident that I could deal efficiently with unexpected events.

a. Strongly agree b. Agree

c. Disagree d. Strongly disagree

5. Thanks to my resourcefulness, I know how to handle unforeseen situations.

a. Strongly agree b. Agree

c. Disagree d. Strongly disagree

6. I can solve most problems if I invest the necessary effort.

a. Strongly agree b. Agree

c. Disagree d. Strongly disagree

7. I can remain calm when facing difficulties because I can rely on my coping abilities.

a. Strongly agree b. Agree

c. Disagree d. Strongly disagree

8. When I am confronted with a problem, I can usually find several solutions.

a. Strongly agree b. Agree

c. Disagree d. Strongly disagree

9. If I am in trouble, I can usually think of a solution
 a. Strongly agree b. Agree

c. Disagree d. Strongly disagree

10. I can usually handle whatever comes my way.

a. Strongly agree b. Agree

c. Disagree d. Strongly disagree

Rosenberg's Self-Esteem Questionnaire (SET)

1. I feel that I am a person of worth, at least on an equal plane with others.

a. Strongly agree b. Agree

c. Disagree d. Strongly disagree

2. I feel that I have a number of good qualities.

a. Strongly agree b. Agree

c. Disagree d. Strongly disagree

3. All in all, I am inclined to feel that I am a failure.

a. Strongly agree b. Agree

c. Disagree d. Strongly disagree

4. I am able to do things as well as most other people.

a. Strongly agree b. Agree

c. Disagree d. Strongly disagree

5. I feel I do not have much to be proud of.

a. Strongly agree b. Agree

c. Disagree d. Strongly disagree

6. I take a positive attitude toward myself.

a. Strongly agree b. Agree

c. Disagree d. Strongly disagree

7. On the whole, I am satisfied with myself.

a. Strongly agree b. Agree

c. Disagree d. Strongly disagree

8. I wish I could have more respect for myself.

a. Strongly agree b. Agree

c. Disagree d. Strongly disagree

9. I certainly feel useless at times.

a. Strongly agree b. Agree

c. Disagree d. Strongly disagree

10. At times I think I am no good at all.

a. Strongly agree b. Agree

c. Disagree d. Strongly disagree

Connor-Davidson Resilience Scale (RE)

1. Able to adapt to change.

A Never B Rarely C Sometimes D Often E Always

2. Close and secure relationships.

A Never B Rarely C Sometimes D Often E Always

3. Sometimes fate or God can help.

A Never B Rarely C Sometimes D Often E Always

4. Can deal with whatever comes.

A Never B Rarely C Sometimes D Often E Always

5. Past success gives confidence for new challenge.

A Never B Rarely C Sometimes D Often E Always

6. See the humorous side of things.

A Never B Rarely C Sometimes D Often E Always

7. Coping with stress strengthens.

A Never B Rarely C Sometimes D Often E Always

8. Tend to bounce back after illness or hardship.

A Never B Rarely C Sometimes D Often E Always

9. Things happen for a reason.

A Never B Rarely C Sometimes D Often E Always

10. Best effort no matter what.

A Never B Rarely C Sometimes D Often E Always

11. You can achieve your goals.

A Never B Rarely C Sometimes D Often E Always

12. When things look hopeless, I don't give up.

A Never B Rarely C Sometimes D Often E Always

13. Know where to turn for help.

A Never B Rarely C Sometimes D Often E Always

14. Under pressure, focus and think clearly.

A Never B Rarely C Sometimes D Often E Always

15. Prefer to take the lead in problem solving.

A Never B Rarely C Sometimes D Often E Always

16. Not easily discouraged by failure.

A Never B Rarely C Sometimes D Often E Always

17. Think of self as strong person.

A Never B Rarely C Sometimes D Often E Always

18. Make unpopular or difficult decisions.

A Never B Rarely C Sometimes D Often E Always

19. Can handle unpleasant feelings.

A Never B Rarely C Sometimes D Often E Always

20. Have to act on a hunch.

A Never B Rarely C Sometimes D Often E Always

21. Strong sense of purpose.

A Never B Rarely C Sometimes D Often E Always

22. In control of your life.

A Never B Rarely C Sometimes D Often E Always

23. I like challenges.

A Never B Rarely C Sometimes D Often E Always

24. You work to attain your goals.

A Never B Rarely C Sometimes D Often E Always

25. Proud in your achievements.

A Never B Rarely C Sometimes D Often E Always

Hamilton Anxiety Rating Scale (HRA)

1. Anxious mood: Worries, anticipation of the worst, fearful anticipation, irritability.

A Not present B Mild C Moderate D Severe E Very severe

2. Tension: Feelings of tension, fatigability, startle response, moved to tears easily, trembling, feelings of restlessness, inability to relax.

A Not present B Mild C Moderate D Severe E Very severe

3. Fears: Of dark, of strangers, of being left alone, of animals, of traffic, of crowds.

A Not present B Mild C Moderate D Severe E Very severe

4. Insomnia: Difficulty in falling asleep, broken sleep, unsatisfying sleep and fatigue on waking, dreams, nightmares, night terrors.

A Not present B Mild C Moderate D Severe E Very severe

5. Intellectual: Difficulty in concentration, poor memory.

A Not present B Mild C Moderate D Severe E Very severe

6. Depressed mood: Loss of interest, lack of pleasure in hobbies, depression, early waking,

diurnal swing.

A Not present B Mild C Moderate D Severe E Very severe

7. Somatic (muscular): Pains and aches, twitching, stiffness, myoclonic jerks, grinding of teeth, unsteady voice, increased muscular tone.

A Not present B Mild C Moderate D Severe E Very severe

8. Somatic (sensory): Tinnitus, blurring of vision, hot and cold flushes, feelings of weakness, pricking sensation.

A Not present B Mild C Moderate D Severe E Very severe

9. Cardiovascular symptoms: Tachycardia, palpitations, pain in chest, throbbing of vessels, fainting feelings, missing beat.

A Not present B Mild C Moderate D Severe E Very severe

10. Respiratory symptoms: Pressure or constriction in chest, choking feelings, sighing, dyspnea.

A Not present B Mild C Moderate D Severe E Very severe

11. Gastrointestinal symptoms: Difficulty in swallowing, wind abdominal pain, burning sensations, abdominal fullness, nausea, vomiting, borborygmi, looseness of bowels, loss of weight, constipation.

A Not present B Mild C Moderate D Severe E Very severe

12. Genitourinary symptoms: Frequency of micturition, urgency of micturition, amenorrhea, menorrhagia, development of frigidity, premature ejaculation, loss of libido, impotence.

A Not present B Mild C Moderate D Severe E Very severe

13. Autonomic symptoms: Dry mouth, flushing, pallor, tendency to sweat, giddiness, tension

headache, raising of hair.

A Not present B Mild C Moderate D Severe E Very severe

14. Behavior at interview: Fidgeting, restlessness or pacing, tremor of hands, furrowed brow, strained face, sighing or rapid respiration, facial pallor, swallowing, etc.

A Not present B Mild C Moderate D Severe E Very severe

IPAQ Long Questionnaire (HRA)

Please think about the activities you do at work, as part of your house and yard work, to get from place to place, and in your spare time for recreation, exercise or sport.

Think about all the vigorous and moderate activities that you did in the last 7 days. (Vigorous physical activities refer to activities that take hard physical effort and make you breathe much harder than normal. Moderate activities refer to activities that take moderate physical effort and make you breathe somewhat harder than normal.)

PART 1: JOB-RELATED PHYSICAL ACTIVITY

1. Do you currently have a job or do any unpaid work outside your home?

- Yes
- No → Skip to PART 2: TRANSPORTATION

2. During the last 7 days, on how many days did you do vigorous physical activities like heavy lifting, digging, heavy construction, or climbing up stairs as part of your work? Think about only those physical activities that you did for at least 10 minutes at a time.

days per week

- No vigorous job-related physical activity → Skip to question 4

3. How much time did you usually spend on one of those days doing vigorous physical activities as part of your work?

_____ hours per day

_____ minutes per day → Skip to question 6

4. Again, think about only those physical activities that you did for at least 10 minutes at a time. During the last 7 days, on how many days did you do moderate physical activities like carrying light loads as part of your work? Please do not include walking.

_____ days per week

- No moderate job-related physical activity → Skip to question 6

5. How much time did you usually spend on one of those days doing moderate physical activities as part of your work?

_____ hours per day

_____ minutes per day

6. During the last 7 days, on how many days did you walk for at least 10 minutes at a time as part of your work? Please do not count any walking you did to travel to or from work.

_____ days per week

- No job-related walking → Skip to PART 2: TRANSPORTATION

7. How much time did you usually spend on one of those days walking as part of your work?

_____ hours per day

_____ minutes per day

PART 2: TRANSPORTATION PHYSICAL ACTIVITY

8. During the last 7 days, on how many days did you travel in a motor vehicle like a train, bus, car, or tram?

_____ days per week

- No traveling in a motor vehicle → Skip to question 10

9. How much time did you usually spend on one of those days traveling in a train, bus, car, tram, or other kind of motor vehicle?

_____ hours per day

_____ minutes per day

10. During the last 7 days, on how many days did you bicycle for at least 10 minutes at a time to go from place to place?

_____ days per week

- No bicycling from place to place → Skip to question 12

11. How much time did you usually spend on one of those days to bicycle from place to place?

_____ hours per day

_____ minutes per day

12. During the last 7 days, on how many days did you walk for at least 10 minutes at a time to go from place to place?

_____ days per week

- No walking from place to place → Skip to PART 3: HOUSEWORK, HOUSE MAINTENANCE, AND CARING FOR FAMILY

13. How much time did you usually spend on one of those days walking from place to place?

_____ hours per day

_____ minutes per day

PART 3: HOUSEWORK, HOUSE MAINTENANCE, AND CARING FOR FAMILY

This section is about some of the physical activities you might have done in the last 7 days in and around your home, like housework, gardening, yard work, general maintenance work, and caring for your family.

14. Think about only those physical activities that you did for at least 10 minutes at a time. During the last 7 days, on how many days did you do vigorous physical activities like heavy lifting, chopping wood, shoveling snow, or digging in the garden or yard?

_____ days per week

- No vigorous activity in garden or yard → Skip to question 16

15. How much time did you usually spend on one of those days doing vigorous physical activities in the garden or yard?

_____ hours per day

_____ minutes per day

16. Again, think about only those physical activities that you did for at least 10 minutes at a time. During the last 7 days, on how many days did you do moderate activities like carrying light loads, sweeping, washing windows, and raking in the garden or yard?

_____ days per week

- No moderate activity in garden or yard → Skip to PART 4: RECREATION, SPORT, AND LEISURE-TIME PHYSICAL ACTIVITY

17. How much time did you usually spend on one of those days doing moderate physical activities inside your home?

_____ hours per day

_____ minutes per day

PART 4: RECREATION, SPORT, AND LEISURE-TIME PHYSICAL ACTIVITY

18. Not counting any walking you have already mentioned, during the last 7 days, on how many days did you walk for at least 10 minutes at a time in your leisure time?

_____ days per week

- No vigorous activity in garden or yard → Skip to question 20

19. How much time did you usually spend on one of those days walking in your leisure time?

_____ hours per day

_____ minutes per day

20. Think about only those physical activities that you did for at least 10 minutes at a time. During the last 7 days, on how many days did you do vigorous physical activities like aerobics, running, fast bicycling, or fast swimming in your leisure time?

_____ days per week

- No vigorous activity in garden or yard → Skip to question 22

21. How much time did you usually spend on one of those days doing vigorous physical activities in your leisure time?

_____ hours per day

_____ minutes per day

22. Again, think about only those physical activities that you did for at least 10 minutes at a time. During the last 7 days, on how many days did you do moderate physical activities like bicycling at a regular pace, swimming at a regular pace, and doubles tennis in your leisure time?

_____ days per week

- No moderate activity in leisure time → Skip to PART 5: TIME SPENT SITTING

23. How much time did you usually spend on one of those days doing moderate physical activities in your leisure time?

_____ hours per day

_____ minutes per day

PART 5: TIME SPENT SITTING

The last questions are about the time you spend sitting while at work, at home, while doing course work and during leisure time. This may include time spent sitting at a desk, visiting friends, reading or sitting or lying down to watch television.

24. During the last 7 days, how much time did you usually spend sitting on a weekday?

_____ hours per day

_____ minutes per day

25. During the last 7 days, how much time did you usually spend sitting on a weekend day?

_____ hours per day

_____ minutes per day

PART 6: SLEEPING TIME

24. During the last 7 days, how much time did you usually spend sleeping on a weekday?

_____ hours per day

_____ minutes per day

25. During the last 7 days, how much time did you usually spend sleeping on a weekend day?

_____ hours per day

_____ minutes per day
