# Supplementary material for: How to reduce anxiety symptoms through individual sport in youth: A longitudinal study over 8-month observation
Source: SAGE Open Med. 2024 Jun 17;12:20503121241258736. doi: 10.1177/20503121241258736 (PMC11185022; doi:10.1177/20503121241258736)
Supplement: sj-docx-2-smo-10.1177_20503121241258736 – Supplemental material for How to reduce anxiety symptoms through individual sport in youth: A longitudinal study over 8-month observation [file sj-docx-2-smo-10.1177_20503121241258736.docx]

**Table 1 Sample characteristics**

|  |  | **Male** | | | | | **Female** | | | | |  |  |  | **Overall** | | | | |
| --- | --- | --- | --- | --- | --- | --- | --- | --- | --- | --- | --- | --- | --- | --- | --- | --- | --- | --- | --- |
| **Wave 1**  **at Baseline** |  | **N** | **Mean** | **SD** | **Range** | | **N** | **Mean** | **SD** | **Range** | | **F** | **p** | **η_p_^2^** | **N** | **Mean** | **SD** | **Range** | |
|  | **Age** | 190 | 19.08 | 0.87 | 17 | 22 | 72 | 18.93 | 0.88 | 18 | 22 | 1.44 | .218 | .006 | 262 | 19.04 | 0.87 | 17 | 22 |
|  | **BMI** | 191 | 21.27 | 3.91 | 14.73 | 38.88 | 73 | 20.98 | 3.90 | 16.25 | 33.00 | 0.28 | .600 | .001 | 264 | 21.19 | 3.90 | 14.73 | 38.88 |
|  | **Self-efficacy** | 191 | 26.53 | 4.39 | 16 | 39 | 73 | 25.07 | 4.32 | 15 | 35 | 5.93 | .016 | .021 | 264 | 26.13 | 4.41 | 15 | 39 |
|  | **Self-esteem** | 191 | 28.27 | 4.66 | 17 | 40 | 73 | 27.81 | 4.47 | 18 | 39 | 0.54 | .465 | .001 | 264 | 28.14 | 4.60 | 17 | 40 |
|  | **Resilience** | 191 | 83.32 | 11.32 | 49 | 117 | 73 | 79.03 | 10.31 | 57 | 109 | 7.97 | .005 | .026 | 264 | 82.13 | 11.19 | 49 | 117 |
|  | **Anxiety** | 191 | 22.57 | 7.64 | 14 | 56 | 73 | 24.27 | 7.55 | 14 | 44 | 2.64 | .105 | .008 | 264 | 23.04 | 7.64 | 14 | 56 |
|  |  |  | **Proportion** | |  |  |  | **Proportion** | |  |  | **x^2^** | **p** | **w** |  | **Proportion** | |  |  |
|  | **PA levels** | 191 | 100% | |  |  | 73 | 100% | |  |  | 5.42 | .066 | .143 | 264 | 100% | |  |  |
|  | **inactive** | 173 | 90.6% | |  |  | 72 | 98.6% | |  |  |  |  |  | 245 | 92.8% | |  |  |
|  | **insufficiently active** | 10 | 5.2% | |  |  | 0 | 0.0% | |  |  |  |  |  | 10 | 3.8% | |  |  |
|  | **active** | 8 | 4.2% | |  |  | 1 | 1.4% | |  |  |  |  |  | 9 | 3.4% | |  |  |
| **Wave 2**  **at 8 Months** |  | **N** | **Mean** | **SD** | **Range** | | **N** | **Mean** | **SD** | **Range** | | **F** | **p** | **η_p_^2^** | **N** | **Mean** | **SD** | **Range** | |
|  | **Age** | 133 | 19.03 | 0.90 | 17 | 22 | 30 | 18.8 | 0.76 | 18 | 20 | 1.66 | .198 | .010 | 163 | 18.99 | 0.882 | 17 | 22 |
|  | **BMI** | 133 | 20.89 | 3.40 | 16.00 | 38.88 | 30 | 20.67 | 3.42 | 17.22 | 31.00 | 0.05 | .751 | .000 | 163 | 20.85 | 3.39 | 16.00 | 38.88 |
|  | **Self-efficacy** | 133 | 27.68 | 4.49 | 17 | 40 | 30 | 25.57 | 4.25 | 18 | 37 | 5.43 | .020 | .033 | 163 | 27.29 | 4.509 | 17 | 40 |
|  | **Self-esteem** | 133 | 28.92 | 4.00 | 17 | 40 | 30 | 28.63 | 3.85 | 18 | 40 | 0.07 | .724 | .000 | 163 | 28.87 | 3.964 | 17 | 40 |
|  | **Resilience** | 133 | 81.76 | 15.23 | 0 | 117 | 30 | 81.47 | 12.89 | 52 | 118 | 0.00 | .922 | .000 | 163 | 81.71 | 14.793 | 0 | 118 |
|  | **Anxiety** | 133 | 22.86 | 8.48 | 0 | 49 | 30 | 25.5 | 9.69 | 14 | 46 | 2.34 | .136 | .014 | 163 | 23.35 | 8.743 | 0 | 49 |
|  |  |  | **Proportion** | |  |  |  | **Proportion** | |  |  | **x^2^** | **p** | **w** |  | **Proportion** | |  |  |
|  | **PA levels** | 133 | 100% | |  |  | 30 | 100% | |  |  | 3.59 | .166 | .148 | 163 | 100% | |  |  |
|  | **inactive** | 59 | 44.4% | |  |  | 19 | 63.3% | |  |  |  |  |  | 78 | 47.9% | |  |  |
|  | **insufficiently active** | 69 | 51.9% | |  |  | 10 | 33.3% | |  |  |  |  |  | 79 | 48.5% | |  |  |
|  | **active** | 5 | 3.8% | |  |  | 1 | 3.3% | |  |  |  |  |  | 6 | 6.0% | |  |  |
|  |  |  |  |  |  |  |  |  |  |  |  | **F** | **p** | **η_p_^2^** |  |  |  |  |  |
|  | **Table Tennis Performance** | 133 | 34.67 | 4.70 | 0.00 | 40.00 | 30 | 35.73 | 2.86 | 28.00 | 40.00 | 1.48 | .236 | .009 | 163 | 34.87 | 4.43 | 0 | 40 |
|  | **Sedentary Hours (week)** | 133 | 9.72 | 6.27 | 0.00 | 25.00 | 30 | 10.19 | 6.65 | 0.00 | 28.00 | 0.19 | .712 | .001 | 163 | 9.81 | 6.32 | 0 | 28 |
|  | **Sleeping Hours (day)** | 133 | 6.42 | 2.71 | 0.00 | 10.00 | 30 | 6.47 | 3.39 | 0.00 | 10.00 | 0.01 | .937 | .000 | 163 | 6.43 | 2.84 | 0 | 10 |

**Table 2 Correlation Matrix**

|  | **1. Age** | **2** | **3** | **4** | **5** | **6** | **7** | **8** | **9** | **10** | **11** | **12** | **13** | **14** | **15** | **16** | **17** | **18** |
| --- | --- | --- | --- | --- | --- | --- | --- | --- | --- | --- | --- | --- | --- | --- | --- | --- | --- | --- |
| **2. BMI** | rs= - .035  ps= .655 |  |  |  |  |  |  |  |  |  |  |  |  |  |  |  |  |  |
| **3. Self-efficacy Wave 1** | rs= - .054  ps= .496 | rs= - .02  ps= .802 |  |  |  |  |  |  |  |  |  |  |  |  |  |  |  |  |
| **4. Self-efficacy Wave 2** | rs= - .184  ps= .019 | rs= - .04  ps= .615 | rs= .323  ps<.001 |  |  |  |  |  |  |  |  |  |  |  |  |  |  |  |
| **5. Δself-efficacy** | rs= - .118  ps= .135 | rs= - .019  ps= .814 | rs= - .549  ps<.001 | rs= .614  ps<.001 |  |  |  |  |  |  |  |  |  |  |  |  |  |  |
| **6. Self-esteem**  **Wave 1** | rs= - .243  ps= .002 | rs= .019  ps= .808 | rs= .33  ps<.001 | rs= .178  ps= .023 | rs= - .118  ps= .132 |  |  |  |  |  |  |  |  |  |  |  |  |  |
| **7. Self-esteem**  **Wave 2** | rs= - .198  ps= .011 | rs= .033  ps= .673 | rs= .28  ps<.001 | rs= .577  ps<.001 | rs= .276  ps<.001 | rs= .37  ps<.001 |  |  |  |  |  |  |  |  |  |  |  |  |
| **8. ΔSelf-esteem** | rs= .071  ps= .368 | rs= .009  ps= .913 | rs= - .088  ps= .263 | rs= .299  ps<.001 | rs= .338  ps<.001 | rs= - .656  ps<.001 | rs= .458  ps<.001 |  |  |  |  |  |  |  |  |  |  |  |
| **9. Resiliance**  **Wave 1** | rs= - .06  ps= .447 | rs= - .005  ps= .951 | rs= .629  ps<.001 | rs= .379  ps<.001 | rs= - .19  ps= .015 | rs= .508  ps<.001 | rs= .442  ps<.001 | rs= - .126  ps= .108 |  |  |  |  |  |  |  |  |  |  |
| **10. Resiliance**  **Wave 2** | rs= - .067  ps= .395 | rs= .055  ps= .488 | rs= .269  ps<.001 | rs= .581  ps<.001 | rs= .288  ps<.001 | rs= .288  ps<.001 | rs= .65  ps<.001 | rs= .252  ps= .001 | rs= .512  ps<.001 |  |  |  |  |  |  |  |  |  |
| **11. ΔResiliance** | rs= - .025  ps= .749 | rs= .066  ps= .406 | rs= - .222  ps= .004 | rs= .336  ps<.001 | rs= .482  ps<.001 | rs= - .1  ps= .205 | rs= .361  ps<.001 | rs= .389  ps<.001 | rs= - .26  ps<.001 | rs= .696  ps<.001 |  |  |  |  |  |  |  |  |
| **12. Anxiety**  **Wave 1** | rs= .17  ps= .03 | rs= - .005  ps= .947 | rs= - .189  ps= .016 | rs= - .162  ps= .038 | rs= .014  ps= .857 | rs= - .236  ps= .002 | rs= - .284  ps<.001 | rs= - .005  ps= .949 | rs= - .307  ps<.001 | rs= - .194  ps= .013 | rs= .038  ps= .629 |  |  |  |  |  |  |  |
| **13. Anxiety**  **Wave 2** | rs= .169  ps= .031 | rs= .071  ps= .371 | rs= - .079  ps= .315 | rs= - .355  ps<.001 | rs= - .247  ps= .001 | rs= - .138  ps= .078 | rs= - .411  ps<.001 | rs= - .202  ps= .01 | rs= - .263  ps<.001 | rs= - .299  ps<.001 | rs= - .117  ps= .137 | rs= .567  ps<.001 |  |  |  |  |  |  |
| **14. ΔAnxiety** | rs= .04  ps= .613 | rs= .087  ps= .267 | rs= .084  ps= .289 | rs= - .264  ps<.001 | rs= - .303  ps<.001 | rs= .058  ps= .463 | rs= - .217  ps= .005 | rs= - .232  ps= .003 | rs= - .021  ps= .788 | rs= - .169  ps= .031 | rs= - .172  ps= .028 | rs= - .268  ps<.001 | rs= .642  ps<.001 |  |  |  |  |  |
| **15. PA Hours**  **Wave 1** | rs= .163  ps= .038 | rs= .01  ps= .901 | rs= .103  ps= .192 | rs= .087  ps= .271 | rs= - .009  ps= .909 | rs= - .042  ps= .597 | rs= - .021  ps= .794 | rs= .023  ps= .769 | rs= .161  ps= .04 | rs= .036  ps= .644 | rs= - .093  ps= .236 | rs= - .022  ps= .781 | rs= .02  ps= .798 | rs= .044  ps= .576 |  |  |  |  |
| **16. PA Hours**  **Wave 2** | rs= .162  ps= .039 | rs= .053  ps= .501 | rs= .069  ps= .383 | rs= .075  ps= .34 | rs= .009  ps= .908 | rs= - .03  ps= .705 | rs= - .06  ps= .45 | rs= - .02  ps= .802 | rs= .168  ps= .033 | rs= .001  ps= .987 | rs= - .138  ps= .078 | rs= - .019  ps= .812 | rs= .031  ps= .691 | rs= .054  ps= .493 | rs= .943  ps<.001 |  |  |  |
| **17. Table Tennis Performance** | rs= - .094  ps= .235 | rs= .076  ps= .334 | rs= .100  ps= .206 | rs= .075  ps= .34 | rs= - .017  ps= .833 | rs= .024  ps= .758 | rs= .086  ps= .272 | rs= .047  ps= .551 | rs= .107  ps= .173 | rs= .103  ps= .193 | rs= .026  ps= .745 | rs= - .011  ps= .888 | rs= - .069  ps= .382 | rs= - .07  ps= .373 | rs= - .005  ps= .946 | rs= - .029  ps= .716 |  |  |
| **18. Sedentary Hours** | rs= - .044  ps= .575 | rs= .042  ps= .596 | rs= - .118  ps= .134 | rs= - .212  ps= .007 | rs= - .089  ps= .259 | rs= - .144  ps= .067 | rs= - .135  ps= .087 | rs= .028  ps= .72 | rs= - .069  ps= .381 | rs= - .081  ps= .302 | rs= - .034  ps= .67 | rs= .013  ps= .871 | rs= - .065  ps= .407 | rs= - .088  ps= .262 | rs= .015  ps= .849 | rs= .025  ps= .748 | rs= .141  ps= .072 |  |
| **19. Sleep** | rs= - .007  ps= .934 | rs= .041  ps= .607 | rs= - .145  ps= .065 | rs= - .104  ps= .186 | rs= .029  ps= .714 | rs= - .009  ps= .909 | rs= .009  ps= .909 | rs= .016  ps= .839 | rs= - .035  ps= .66 | rs= .128  ps= .105 | rs= .172  ps= .028 | rs= - .136  ps= .083 | rs= - .123  ps= .117 | rs= - .017  ps= .825 | rs= .057  ps= .471 | rs= .04  ps= .613 | rs= .058  ps= .461 | rs= .337  ps<.001 |
| Sample Size | 163 | 163 | 163 | 163 | 163 | 163 | 163 | 163 | 163 | 163 | 163 | 163 | 163 | 163 | 163 | 163 | 163 | 163 |

**Table 3 Multiple regression analysis controlling for covariates**

| **Dependent** | **Predictor** | **B** | **SE** | **t** | **p** | **Tolerance** | **VIF** |
| --- | --- | --- | --- | --- | --- | --- | --- |
| **ΔAnxiety** | **Δself-efficacy** | -0.43 | 0.18 | -2.43 | .016 | .363 | 2.755 |
|  | **Δself-esteem** | -0.38 | 0.15 | -2.46 | .015 | .438 | 2.283 |
|  | **Δsedentary** | -0.17 | 0.09 | -1.99 | .049 | .916 | 1.092 |
|  | **Coviarates** | **B** | **SE** | **t** | **p** | **Tolerance** | **VIF** |
|  | **Age** | .42 | .65 | .65 | .516 | .856 | 1.169 |
|  | **BMI** | .20 | .16 | 1.27 | .205 | .990 | 1.011 |
|  | **Sex** | 1.90 | 1.41 | 1.35 | .180 | .928 | 1.078 |
|  | **Self-efficacy at Wave 1** | .01 | .21 | .07 | .946 | .360 | 2.782 |
|  | **Self-esteem at Wave 1** | -.27 | .21 | -1.29 | .198 | .301 | 3.318 |
|  | **Resilience at Wave 1** | -.09 | .07 | -1.20 | .231 | .433 | 2.309 |
|  | **Anxiety at Wave 1** | -.38 | .08 | -4.62 | <.001 | .860 | 1.163 |
|  | **Physical activity at Wave 1** | .59 | .74 | .79 | .431 | .913 | 1.095 |
|  | **Predictor** | **B** | **SE** | **t** | **p** | **Tolerance** | **VIF** |
| **ΔSelf-esteem** | **Table Tennis Performance** | .03 | .09 | .54 | .589 | .960 | 1.042 |
|  | **Sedentary** | -.08 | .06 | -1.38 | .168 | .960 | 1.042 |
|  | **Sleep** | .01 | .14 | .09 | .931 | .944 | 1.059 |
|  | **Coviarates** | **B** | **SE** | **t** | **p** | **Tolerance** | **VIF** |
|  | **Age** | -.52 | .33 | -1.58 | .117 | .893 | 1.12 |
|  | **BMI** | .03 | .08 | .39 | .698 | .997 | 1.003 |
|  | **Sex** | -.42 | .72 | -.58 | .562 | .969 | 1.032 |
|  | **Self-efficacy at Wave 1** | .00 | .08 | .00 | 1.000 | .604 | 1.655 |
|  | **Self-esteem at Wave 1** | -.88 | .07 | -12.34 | <.001 | .683 | 1.464 |
|  | **Resilience at Wave 1** | .12 | .04 | 3.26 | .001 | .468 | 2.138 |
|  | **Anxiety at Wave 1** | -.07 | .04 | -1.76 | .081 | .879 | 1.138 |
|  | **PA at Wave 1** | -.31 | .39 | -.80 | .426 | .923 | 1.084 |

**Table 4 Simple slop analysis of self-esteem moderating effect of table tennis**

| **Model items** | **β** | **SE** | **t** | **p** |  |  |
| --- | --- | --- | --- | --- | --- | --- |
| **Table Tennis Performance** | -.72 | .25 | -2.83 | .005 |  |  |
| **ΔSelf-esteem** | -15.02 | 4.49 | -3.35 | .001 |  |  |
| **ΔSelf-esteem×Table Tennis Performance** | .34 | .13 | 2.70 | .008 |  |  |
| **Coviarates** | **β** | **SE** | **t** | **p** | **Tolerance** | **VIF** |
| **Age** | .50 | .63 | .80 | .428 | .860 | 1.163 |
| **BMI** | .26 | .15 | 1.68 | .095 | .988 | 1.012 |
| **SEX** | 3.13 | 1.35 | 2.31 | .022 | .956 | 1.046 |
| **Self-efficacy at Wave 1** | .32 | .16 | 2.03 | .044 | .582 | 1.72 |
| **Self-esteem at Wave 1** | -.24 | .16 | -1.57 | .118 | .330 | 3.028 |
| **Resilience at Wave 1** | -.10 | .07 | -1.49 | .139 | .432 | 2.314 |
| **Anxiety at Wave 1** | -.31 | .08 | -3.93 | .000 | .836 | 1.196 |
| **PA at Wave 1** | .77 | .72 | 1.06 | .289 | .912 | 1.097 |
| **Sedentary** | -.16 | .09 | -1.78 | .076 | .825 | 1.212 |
| **Sleep** | .07 | .20 | .36 | .717 | .832 | 1.202 |
